# The statistical mechanics of human weight change

**DOI:** 10.1371/journal.pone.0189795

**Published:** 2017-12-18

**Authors:** John C. Lang, Hans De Sterck, Daniel M. Abrams

**Affiliations:** 1 Department of Communication Studies, University of California Los Angeles, Los Angeles, California, United States of America; 2 School of Mathematical Sciences, Monash University, Clayton, Victoria, Australia; 3 Department of Engineering Sciences and Applied Mathematics, Northwestern University, Evanston, IL, United States of America; 4 Northwestern Institute for Complex Systems, Northwestern University, Evanston, IL, United States of America; University of California Irvine, UNITED STATES

## Abstract

Over the past 35 years there has been a near doubling in the worldwide prevalence of obesity. Body Mass Index (BMI) distributions in high-income societies have increasingly shifted rightwards, corresponding to increases in average BMI that are due to well-studied changes in the socioeconomic environment. However, in addition to this shift, BMI distributions have also shown marked changes in their particular shape over time, exhibiting an ongoing right-skewed broadening that is not well understood. Here, we compile and analyze the largest data set so far of year-over-year BMI changes. The data confirm that, on average, heavy individuals become lighter while light individuals become heavier year-over-year, and also show that year-over-year BMI evolution is characterized by fluctuations with a magnitude that is linearly proportional to BMI. We find that the distribution of human BMIs is intrinsically dynamic—due to the short-term variability of human weight—and its shape is determined by a balance between deterministic drift towards a natural set point and diffusion resulting from random fluctuations in, e.g., diet and physical activity. We formulate a stochastic mathematical model for BMI dynamics, deriving a theoretical shape for the BMI distribution and offering a mechanism that may explain the right-skewed broadening of BMI distributions over time. An extension of the base model investigates the hypothesis that peer-to-peer social influence plays a role in BMI dynamics. While including this effect improves the fit with the data, indicating that correlations in the behavior of individuals with similar BMI may be important for BMI dynamics, testing social transmission against other plausible unmodeled effects and interpretations remains the subject of future work. Implications of our findings on the dynamics of BMI distributions for public health interventions are discussed.

## Introduction

**Fig 1:**
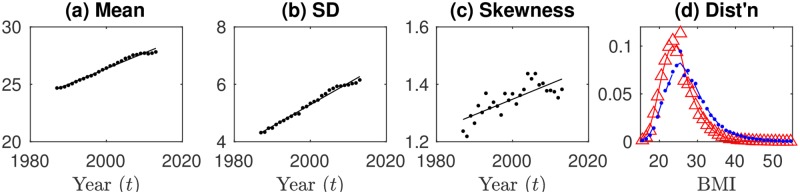
Empirical mean, standard deviation, and skewness of the BMI distribution for BRFSS survey data. BMI mean, SD, and skewness have steadily increased over the course of the obesity epidemic, with growth tempered in recent years. (a)-(c): dots show data points, lines show show regression fits; (d): probability distributions for BMI in 1991 (red triangles show binned data, red solid curve shows smoothed histogram) and 2011 (blue circles show binned data, blue dashed curve shows smoothed histogram).

Obesity is a risk factor for many chronic illnesses [1–3], and the obesity epidemic has become one of the major public health concerns of our time [4, 5]. Understanding who becomes obese and why has direct implications in the quest for adequate public health interventions, for example, to determine whether high-risk individuals or the whole population should be targeted [6, 7]. The Body Mass Index (BMI), defined as the mass (in kilograms) divided by the height (in meters) squared, is a standard measure of relative body weight used to classify individuals as underweight (BMI ≤ 18.5), normal weight (18.5 < BMI ≤ 25), overweight (25 < BMI ≤ 30), or obese (BMI > 30). The distribution of BMIs in high-income societies is right-skewed (i.e., skewed towards the high-BMI side) and the mean and standard deviation (SD) have steadily increased over time [8–10]. The increasing mean of the distribution is the result of ongoing society-wide shifts in lifestyle and nutrition, but the causes of the right-skewness and broadening in time are debated [8, 10–12]. Fig 1, using national health survey data in the United States provided by the Behavioural Risk Factor Surveillance System (BRFSS) [13], illustrates that BMI mean and SD have both steadily grown since at least 1987 while the obesity epidemic was running its course (with tempered growth in more recent years) [4, 5, 8, 10]. The third panel shows that the skewness of the distribution (where positive skewness mean skewness to the right) has also steadily risen. The fourth panel shows that the distribution has indeed shifted markedly to the right between, e.g., 1991 and 2011, and that the distribution has broadened especially on the high-BMI right side (see S1 Video for BRFSS BMI distributions from 1987–2013). Recent results show that this right-skewed broadening of the distribution is not driven by socioeconomic and demographic factors since it occurs equally within social and demographic subgroups [10]. Therefore, alternative explanations for the broadening have been put forward that include variations in genetic susceptibility to obesogenic environmental factors [10, 14], and the “runaway train” theory that BMI distributions are right-skewed because high-BMI individuals become subject to a vicious self-reinforcing cycle of weight gain [11, 12]. Also, uncertainty remains over the importance of external factors such as microbial influence [15] or peer influence [16–19].

Here, we present a novel data set of BMI measurements for more than 750,000 individuals receiving Chicago-area medical services [20], and a new mechanistic mathematical model for BMI dynamics that is informed by the trends we identify in the data. We analyze year-over-year BMI changes in the new data set and in a smaller existing survey data set, which leads to observations on how the average and standard deviation of year-over-year changes in BMI vary as a function of BMI. The data provides strong indications that human BMI distributions are determined by a balance between deterministic drift towards a natural set point, and diffusion resulting from random fluctuations in, e.g., diet and physical activity. The data shows that low-BMI individuals on average increase their weight year-over-year, and high-BMI individuals decrease their weight, with the increase/decrease being approximately linear in BMI. We also find empirically that year-over-year BMI evolution is characterized by fluctuations with a magnitude that is linearly proportional to BMI. These observations indicate that fluctuations are an important factor in BMI dynamics, and we use this finding as an essential part of the stochastic mathematical model we propose, in which the aggregate influence of fluctuations is modeled as a random effect.

The effects we observe are in some sense expected: if human weight is dynamic on short timescales and population distributions are in quasi-equilibrium at any given time, then BMI distributions have to be characterized by a balance between drift towards the center of the distribution and diffusion that is an aggregate effect of multifactorial perturbations. However, as far as we are aware these effects have not been studied and carefully quantified in large data sets before, and their significance for the shape of BMI distributions has not been recognized previously. Informed by the observations, in particular, that fluctuations are linearly proportional to BMI, we formulate a simple stochastic model for BMI dynamics. Our model provides some understanding of the observed drift-diffusion effects by relating them to known processes from the obesity literature and to drift-diffusion mechanisms that are familiar from statistical mechanics applications in the physical sciences. The model then naturally leads to a new mechanistic explanation for the observed right-skewed broadening of BMI distributions over time, the cause of which is the subject of ongoing debate with implications for intervention strategies. It can be noted here that understanding how this right-skewed broadening occurs is also important because the broadening implies that the standard measure of obesity (BMI > 30) may show larger increases, than, for example, increases in average BMI.

More generally, there is currently no quantitative mathematical model describing how individuals change weight over time, and how the behavior of individuals influences properties of the distribution. Our model proposes a stochastic mechanism that is directly informed by the dynamical effects we observe in the data, and can be related to effects that were previously described in a qualitative manner in the BMI literature. The model closely replicates BMI data from three independent data sets at both the level of individuals and populations. We also consider an extended model to investigate the hypothesis that peer-to-peer social influence plays a role in BMI dynamics. We note that our model differs from previous statistical studies [16, 17, 19, 21] that investigate the role of social and peer influences in that we propose an actual mechanism through which social and peer influences can affect dynamics of the BMI. Our model also differs from previous compartmental [22, 23] and network [24, 25] mathematical models in that our model proposes specific mechanisms and a derived BMI distribution that are rooted in the dynamical effects we observe in the data. Similar to important population-level models in mathematical biology such as the Susceptible-Infected-Recovered (SIR) epidemiological model of Kermack and McKendrick [26], our model is simple in that it models the entire population without regard to factors like age, gender, etc. While such factors are undeniably important in understanding key aspects of the obesity epidemic, simple population-level models can, like SIR, play an important role in identifying and quantifying major effects at play across the population. The focus of this paper is to formulate such a population-level mathematical model for BMI dynamics, grounded in observational data. At the same time, in S1 Appendix we do confirm that the population-level effects we observe and model are also present across differentiated age and gender categories.

The remainder of this paper is organized as follows. In the Data section we present our new BMI data set and report on our findings regarding drift and diffusion in BMI distributions. Informed by the dynamical effects identified in the new BMI data, we propose in the Methods and mathematical models section a new stochastic mathematical model of BMI evolution for individuals and populations, deriving a new theoretical shape for BMI distributions. In the Discussion section we discuss the implications of our findings in offering a mechanism to explain the ongoing right-skewed broadening over time of BMI distributions in high-income societies, and some implications for the debate on whether high-risk individuals or the whole population should be targeted in public health interventions. Additional details on data sets and the mathematical model are provided in S1 Appendix.

## Data

**Fig 2:**
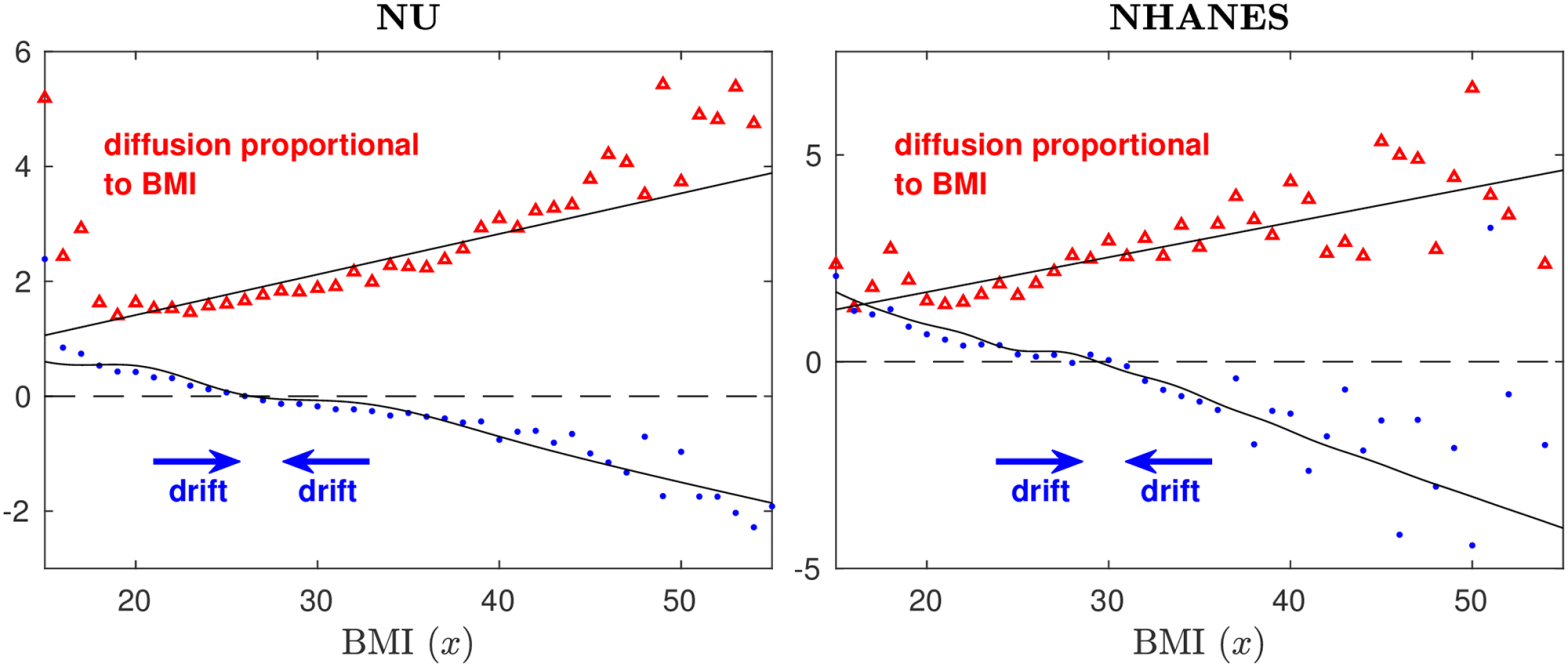
Drift and diffusion in the short-term BMI dynamics of individuals in a human population. The figure shows the average annual change in the BMI of individuals (blue dots), and the standard deviation of the annual change in the BMI of individuals (red triangles), as a function of BMI, for data from our new large NU data set (left panel; 121,574 measurements for 2011) and from the publicly available NHANES survey data set (right panel; 5,624 measurements for 2011–2012). The plots are obtained by binning empirical BMI differences. The blue curves (dots) show that low-BMI individuals on average increase their weight year-over-year, while high-BMI individuals decrease their weight on average, and the dependence on BMI is approximately linear. The red curves (triangles) show that the standard deviation of annual BMI changes, which results from natural short-term fluctuations in an individual’s BMI that may be due to variations in diet or physical activity, increases approximately linearly as a function of BMI. These results establish that BMI dynamics feature a *drift* towards a set point, and a *diffusion* that is proportional to the BMI. The black curves are the curves of best fit for all data years to our mathematical models for the drift term (Eq (2), including social effects) and for the diffusion amplitude (Eq (10)), as discussed in the Methods and mathematical models section. Fig A of S1 Appendix repeats this analysis for the NU and NHANES BMI data split up by age range and by gender, confirming the drift-diffusion dynamics identified here. Fig D of S1 Appendix repeats this analysis for the entire data set over all years, confirming the nearly linear relations observed here.

For this work we require two different types of BMI data: population-level and individual-level. At the population level we consider empirical BMI distributions over a population. We compute empirical BMI distributions from three independently collected data sets: our new data set of medical records for Chicago-area patients of the Northwestern Medicine system of hospitals and clinics (NU) that we make freely available [20], and two publicly available data sets that derive from national health surveys in the United States, the National Health and Nutrition Examination Survey (NHANES) [27], and the BRFSS [13]. At the level of individuals we consider the average change in individuals’ BMIs over time and the standard deviation in the changes in individuals’ BMIs, both as a function of BMI (see Fig 2 and Fig A of S1 Appendix). We can compute the temporal change in individuals’ BMIs from two independently collected data sets: the new NU and the existing NHANES data sets. Our study and model focus on BMI changes of individuals over short timescales, and in practice a suitable timescale for which data on BMI change is available is of the order of about a year, since multiple measurements typically exist for patients visiting hospitals on the time scale of a year, and health survey data also often provide information on annual changes.

### New data set: Northwestern Medicine medical records

As part of this study, we compile and present analysis of an entirely new BMI data set more abundant than any previously reported. BMI measurements calculated from anonymized medical records for more than 750,000 patients of the Northwestern Medicine system of hospitals and clinics are considered from 1997 through 2014, with the majority of records coming from later years. We calculate BMI from weight and height data for individuals in this data set that are at least 18 years of age. We use these data to compute the empirical BMI distribution for each year. In addition, we are able to calculate the change in BMI over one year for all individuals with patient records in consecutive years. Specifically, we extract from the Northwestern Medicine medical record 1,017,518 measurements of year-over-year BMI change for 329,543 distinct individuals. We note that this data set provides the most abundant source of individual level data. However, one caveat is that these data do not form a fully representative sample of the population. For example, since these data are comprised of medical records they may be biased toward less healthy individuals, subject to self-selection effects, etc. For this reason, we carefully vet all our results and findings by cross-comparison with the NHANES and BRFSS survey data, which can be assumed to be more representative of the US population. Nevertheless, our new NU data are extremely valuable since they were recorded during actual physical exams (unlike some of the survey interview data which were self-reported). They represent the largest data set of its type and allow us to conduct more detailed studies. For additional details on the NU data, see Section S1.1.1 of S1 Appendix.

### Publicly available NHANES and BRFSS survey data

In S1 Appendix Sections S1.1.2–3 we describe the publicly available NHANES and BRFSS survey data. NHANES data are available for survey years 1999–2000, 2001–2002, …, 2013–2014, and allow us to consider empirical BMI distributions based on approximately 5,000 adult individuals per year whose weight and height measurements were taken during a physical exam. The NHANES data also provide self-reported change in BMI over the year preceding the survey interview. We consider BRFSS data for survey years from 1987 to 2013. The number of individual records increases from approximately 50,000 in 1987, to more than 400,000 from 2007 onward. Weight and height measurements are self-reported. We use BRFSS data as a third source for empirical BMI distributions, but the BRFSS data does not contain information that allows us to infer annual BMI change for individuals.

### Average and standard deviation of year-over-year BMI changes of individuals

Fig 2 presents novel observations on BMI dynamics: on short timescales of about a year, the BMIs of individuals in a human population show a natural drift *on average* towards the center of the BMI distribution, and show diffusion (resulting from fluctuations due to multifactorial perturbations) with an amplitude that is approximately proportional to the BMI. We demonstrate this for measurements from two independent data sets: our newly compiled large NU data set, compared with the much smaller but publicly available NHANES data set.

The blue dots in Fig 2 give the average annual change in the BMI of individuals as a function of BMI for a representative year (2011–2012 NHANES survey data and NU data for individuals with measurements taken in 2011 and 2012). The averages are taken over bins of empirical BMI differences: BMI differences that originate from a similar starting BMI are placed in the same bin. Specifically, to generate Fig 2 we first compute average and standard deviation of year-over-year BMI differences on the 90-point grid {10.5, 11.5, 12.5, …, 99.5}. For each grid point the average and standard deviation of year-over year BMI differences are taken over the bin containing all BMI differences with initial BMI within $\epsilon =\frac{1}{2}$ of the grid point. For the 2011–2012 NU data there are 121,574 individual BMI difference measurements and each bin (associated with a point in the grid {10.5, 11.5, …, 99.5}) contains on average 1,350 BMI differences. For 2011–2012 NHANES data there are 5,624 individual BMI difference measurements and each bin contains on average 62 BMI differences. Fig A of S1 Appendix repeats this analysis for the NU and NHANES BMI data split up by age range and by gender, confirming the drift-diffusion dynamics identified here. In Section S1.2 of S1 Appendix we explain how we fit the parameters of our stochastic model described in the Methods and mathematical models section to the observed data (black curves in Fig 2).

### Interpretation in terms of a drift-diffusion mechanism

Fig 2 shows the distinctive trend that *on average* low-BMI individuals increase their weight year-over-year, while high-BMI individuals decrease their weight *on average* (blue dots), with the increase/decrease being approximately linear in BMI. This lends quantitative support to the BMI set point hypothesis: the intrinsic dynamics of weight change in healthy adults are thought to follow a “return to equilibrium” pattern where individuals tend to fluctuate about a natural equilibrium, or “set point” [28–30]. The red triangles in Fig 2 show, in a striking manner, that the SD of annual BMI changes increases approximately linearly with BMI. The variation in annual BMI change results from the aggregate in short-term fluctuations that may be due to variations in, e.g., diet and physical activity, and other effects. For the NHANES data, a clear nearly-linear relation can be observed in the SD for a BMI of up to about 35–40, but for larger BMIs the number of data points is small and results become noisy. For the more extensive NU data set, the near-linear relation can be observed up to a BMI of about 45. It has to be noted, though, that for the NU data self-selection effects of return patients who may actively be addressing a high BMI may have an influence. The observed nearly linear relation in the SD over a large part of the BMI range is plausible: higher-BMI individuals are expected to lose or gain more weight when subjected to perturbations such as a diet [28], for biological reasons [8, 12]. For further analysis and comparison, we repeat Fig 2 (with 2011–2012 data) for the entire data set over all years in Fig D of S1 Appendix. Fig D of S1 Appendix confirms, for the entire data set, the nearly linear relations for the annual change and its standard deviation that were identified in Fig 2 for data years 2011–2012. Due to increased data size, the curves for the entire data set are less noisy. Fig D of S1 Appendix also shows that the standard deviation appears to grow faster than linear for large BMIs greater than about 45, both for the NU patient data and the NHANES population data (which is still noisy for the largest BMIs).

While high-BMI individuals decrease their weight *on average*, they are subject to BMI fluctuations with an amplitude (the SD) that is greater than the average decrease in their BMI (Fig 2). The drift towards the center of the BMI distribution is balanced by these fluctuations, and the fluctuations broaden the distribution away from the center. This can be understood in analogy with well-known processes from the physical sciences. For example, a massive Brownian particle under the influence of friction due to collisions with molecules in the surrounding medium [31] follows a deterministic path, but at the scale of large populations the collisions between molecules and Brownian particles can be modeled as random fluctuations. The velocity distribution of the Brownian particles can be described accurately by a balance between deterministic drift towards zero velocity (due to friction) and a stochastic diffusion process that models random noise (as described by the Ornstein-Uhlenbeck process [31]), resulting in a Gaussian velocity distribution at equilibrium. In a similar manner our observations from Fig 2 imply that the BMI distribution is intrinsically dynamic, due to the short-term variability of human weight, and can be described, in first approximation, as the result of a balance between deterministic drift and random diffusion. This is unlike, e.g., the adult height distribution in a human population, which is essentially static on timescales of about a year (because adult height hardly changes) and is nearly normally distributed, as opposed to the strongly skewed distributions that are observed for BMI. We now proceed to describe this drift-diffusion balance for BMI distributions quantitatively using a stochastic mathematical model.

## Methods and mathematical models

We model the temporal evolution of the BMI *x*_*i*_ of an individual *i* by the Langevin equation [31]

$$\begin{array}{c} \frac{dx_{i}}{dt}=a\left(x_{i}\right)+b\left(x_{i}\right)\;\eta \left(t\right)\;, \end{array}$$ (1)

where *t* is time, *a*(*x*_*i*_) is a drift (or advection) term and *b*(*x*_*i*_)*η*(*t*) forms a random diffusion term (*η*(*t*) represents Gaussian white noise). Since the mean of *dx*_*i*_ is given by $E[dx_{i}]=a\left(x_{i}\right)dt$ and the variance of *dx*_*i*_ by $E\left[dx_{i}^{2}\right]-E{\left[dx_{i}\right]}^{2}=b{\left(x_{i}\right)}^{2}dt$, the average of changes in the individual’s BMI per time interval *dt* follows the drift term *a*(*x*), and the SD of BMI changes follows *b*(*x*).

### Modeling drift dynamics

We model the drift term by

$$\begin{array}{c} a\left(x_{i}\right)=k_{I}\;\left(x^{⋆}-x_{i}\right)+k_{S}\;G\left(x_{i},\vec{x};\sigma \right). \end{array}$$ (2)

The first term in Eq (2) represents intrinsic set point dynamics, describing the theory that individuals tend to fluctuate about a natural equilibrium *x*^⋆^ [28–30]. Our observations of mean annual BMI change in Fig 2 suggest a linear relationship with slope *k*_*I*_ ∼ 0.1yr^−1^ as a suitable initial approximation.

In an extension of our basic model we consider the second term of *a*(*x*_*i*_) in Eq (2), which models the extrinsic social influence that individuals may exert on each other, and we base it on the homophily-motivated assumption that individuals interact most strongly with others that are similar [32–34]. We incorporate this effect because our large new data set offers us the opportunity to investigate the hypothesis that peer-to-peer effects influence BMI dynamics [16, 17, 19]. In the second term, *k*_*S*_ is a rate constant and $G(x_{i},\vec{x};\sigma )$ is derived from Gaussian interaction kernels with SD *σ* that model the influence between individual *i* and the other individuals represented by $\vec{x}$, as explained in more detail below.

#### Modeling intrinsic set point dynamics

More specifically, the intrinsic dynamics of return to a set point weight is modeled by assuming exponential decay to equilibrium as

$$\begin{array}{c} \frac{dx_{i}}{dt}=k_{I}\left(x_{i}^{⋆}-x_{i}\right)\;, \end{array}$$ (3)

where $x_{i}^{⋆}$ represents the individual’s BMI set point, and the constant *k*_*I*_ > 0 determines the rate of exponential relaxation to equilibrium weight (note that we assume constant height in adults over time, so changes in BMI—defined as the ratio of weight to height squared—are proportional to weight changes). This set point weight may depend upon many factors including genetics, average exercise and eating habits, etc. Though the set point may vary gradually over the course of an individual’s life, we approximate it as a constant on the shorter time scale over which our model applies. In addition, to obtain tractable models, we assume in most of our approach that individuals have a common set point *x*^⋆^. This is a reasonable first approximation as indicated by the curves of average annual BMI change in Fig 2, which shows that there is a nearly linear variation with an intersection point of the curve that is relatively clearly defined. (Section S1.2.1 of S1 Appendix comments on extending aspects of our model to non-constant set points *x*^⋆^.)

Another way to deduce this same model for intrinsic set point dynamics is to assume that individuals tend to maximize some *individual utility function* *u*_*I*_(*x*) = *u*_*I*_(*x*; *x*^⋆^), which by assumption must have a local maximum when BMI *x* = *x*^⋆^ and can be modeled in first approximation by a quadratic as in

$$\begin{array}{c} u_{I}\left(x\right)=-\frac{1}{2}k_{2}{\left(x-x^{⋆}\right)}^{2}. \end{array}$$ (4)

Assuming that the rate of change of BMI will be proportional to the rate of increase of utility,

$$\begin{array}{c} \frac{dx}{dt}=k_{2}^{\prime }\frac{du_{I}}{dx}\;, \end{array}$$ (5)

we arrive at the same intrinsic dynamics as model (3) (Eqs (3) and (5) are identical when $k_{I}=-k_{2}^{\prime }\;k_{2}$).

#### Modeling extrinsic social influence dynamics

The second term in Eq (2) models the extrinsic, peer-to-peer social part of the drift dynamics. Some theories suggest that individuals can become accustomed to the average BMI of peers under exposure to different peer environments [32, 33] and, to reduce disparity, may adjust their weights [34, 35]. We assume that there exists some *social utility function* $u_{S}\left(x\right)=u_{S}\left(x;{\vec{x}}_{\text{peer}}\right)$ which captures this proposed peer-influence phenomenon: the social utility should peak when an individual reaches a BMI consistent with his or her peer(s), ${\vec{x}}_{\text{peer}}$, where ${\vec{x}}_{\text{peer}}$ is a vector containing the BMIs of the peers. Similarly to the intrinsic dynamics, we expect this utility to be well approximated, for the case of a single peer, by a quadratic function (at least locally) and therefore propose

$$\begin{array}{c} v\left(x;x_{\text{peer}}\right)=-\frac{1}{2}k_{3}{\left(x_{\text{peer}}-x\right)}^{2}\;, \end{array}$$ (6)

where we assume that *k*_3_ > 0 is a constant, and where *x*_peer_ is the BMI of some peer who influences the individual under consideration. When multiple peers simultaneously influence an individual, the net social utility becomes

$$\begin{array}{c} u_{S}\left(x_{i}\right)=u_{S}\left(x_{i};\vec{x}\right)=-\frac{1}{2}k_{3}\sum_{j=1}^{N}A_{ij}{\left(x_{j}-x_{i}\right)}^{2}\;, \end{array}$$

where *N* is the number of individuals in the population, $\vec{x}={\left(x_{1},x_{2},\ldots ,x_{N}\right)}^{T}$, and *A*_*ij*_ represents the strength of social influence of individual *j* on individual *i*. Note that we use *v* to denote the social influence of a single peer and *u* for the cumulative effect of multiple peers.

In order to specify *A*_*ij*_ we make the homophily-motivated assumption that individuals with similar BMI interact more strongly than individuals with different BMI [32–35]. Consistent with this assumption, we choose a Gaussian interaction kernel

$$\begin{array}{c} A_{ij}=\frac{1}{N}\phi_{x_{i},\sigma }(x_{j}), \end{array}$$ (7)

where *N* is the population size, *σ* > 0 is a fixed parameter, and *ϕ*_*μ*,*σ*_(*x*) is the probability density function of a normal random variable with mean *μ* and standard deviation *σ* evaluated at *x*. This has the effect of imposing stronger interaction among more similar individuals.

Combining both the intrinsic and extrinsic aspects of the proposed drift process, we obtain

$$\begin{array}{c} \frac{dx_{i}}{dt}=\frac{d}{dx_{i}}\left(u_{I}\left(x_{i}\right)+u_{S}\left(x_{i}\right)\right)=a\left(x_{i}\right) \end{array}$$ (8)

where

$$\begin{array}{cc} a(x_{i}) & =k_{I}\left(x^{⋆}-x_{i}\right)\\ & +k_{S}\underset{G(x_{i},\vec{x};\sigma )}{\underset{︸}{\sum_{j=1}^{N}[A_{ij}\left(x_{j}-x_{i}\right)-\frac{1}{2}\frac{\partial A_{ij}}{\partial x_{i}}{\left(x_{j}-x_{i}\right)}^{2}]}}, \end{array}$$ (9)

and the constants *k*_*I*_ and *k*_*S*_ = −*k*_3_ set the relative importance of individual versus social factors. Note that the summation in Eq (9) corresponds to $G(x_{i},\vec{x};\sigma )$ in Eq (2).

It has to be noted here that the second term in Eq (9) was motivated by a social transmission interpretation, but more broadly it can be interpreted as an extension of our base model that adds the effect of correlations in the behavior of individuals with similar BMI. One hypothesis that would lead to this kind of correlations is indeed social transmission, but there are other possible effects that may result in such correlations. We mention two examples: food insecurity [36, 37], which may affect individuals in a way that is correlated with their BMI, and gene-environment interactions with certain genetic variants that are more common in people with higher BMIs [38]. We will thus keep these alternative interpretations in mind when discussing our results. Similarly, we emphasize that this is just one possible extension of the basic model, and it is possible that other unmodeled effects are equally or more important.

### Modeling diffusion dynamics

We model the diffusion amplitude *b*(*x*_*i*_) in Eq (1) as follows. Consistent with our observations from Fig 2 that fluctuations in an individual’s BMI are roughly proportional to BMI, we take

$$\begin{array}{c} b\left(x_{i}\right)=\sqrt{k_{b}}\;x_{i}\;, \end{array}$$ (10)

with constant *k*_*b*_ > 0. Note that this is also consistent with the biological expectation that high-BMI individuals tend to lose or gain more weight due to perturbations like a diet [8, 12].

### Fokker-Planck equation and equilibrium distribution

In the limit of large population size *N* → ∞, the aggregate dynamics of individuals described by Langevin Eq (1) are given by the population-level Fokker-Planck equation [31]

$$\begin{array}{c} \frac{\partial p}{\partial t}\left(x,t\right)=-\frac{\partial }{\partial x}\left[p\left(x,t\right)a\left(x\right)\right]+\frac{1}{2}\frac{\partial^{2}}{\partial x^{2}}\left[p\left(x,t\right)b^{2}\left(x\right)\right], \end{array}$$ (11)

where *p*(*x*, *t*) is the probability density function for BMI *x* at time *t*. The correspondence with the Langevin equation is exact when *k*_*S*_ = 0 (no social effects), and we assume that it holds in first approximation otherwise, since social effects are a relatively small correction to the dominant linear trend of the drift term *a*(*x*).

We now derive an analytical solution for the BMI distribution under the simplifying assumption that the BMI distribution is close to equilibrium. We thus obtain a closed-form solution for the theoretical BMI distribution without social effects (*k*_*S*_ = 0 in Eq (2)):

$$\begin{array}{c} p_{eq}^{\left(0\right)}\left(x\right)=c\;x^{-2(k_{I}/k_{b}+1)}\;\exp(-2\frac{k_{I}}{k_{b}}\frac{x^{⋆}}{x}), \end{array}$$ (12)

where *c* is a normalization constant given by

$$\begin{array}{c} c^{-1}={\left(2x^{⋆}\frac{k_{I}}{k_{b}}\right)}^{-2k_{I}/k_{b}-1}\Gamma \left(2k_{I}/k_{b}+1\right), \end{array}$$

and $\Gamma \left(t\right)=\int_{0}^{\infty }x^{t-1}e^{x}dx$ is the Gamma function.

The assumption of quasi-equilibrium is well justified if parameter values in our model drift on a time scale slower than individual equilibration times, which we measure at roughly 7–17 years (based on *k*_*I*_ ∼ 0.06–0.14 from Table A in S1 Appendix). Such an assumption seems reasonable for times before the recent onset of the obesity epidemic; after onset we expect the approximation to be less accurate but that the resulting errors should still be small compared to other sources of error. Further justification that the resulting quasi-stationary distribution is a reasonable approximation is provided in Section S1.2.3 of S1 Appendix and in S1 Video, where we compute numerical solutions to the time-dependent Fokker-Planck equation, fitted to the observed data over all years, and find a good match with the analytic quasi-stationary distribution of Eq (12) fitted year-by-year.

When social effects are included (*k*_*S*_ ≠ 0 in Eq (2)), no closed-form solution exists and the equilibrium distribution must be calculated numerically (see Section S1.2 of S1 Appendix).

We note that since $p_{eq}^{\left(0\right)}\left(x\right)∼x^{-2(k_{I}/k_{b}+1)}$ as *x* → ∞, $p_{eq}^{\left(0\right)}\left(x\right)$ becomes a scale-free (or power law) distribution. Note that the linear assumption of Eq (10) also naturally implies a vital property of the equilibrium distribution in our model, namely, that the probability is confined to positive BMIs. Indeed, diffusion of probability is halted at *x* = 0.

## Results

**Fig 3:**
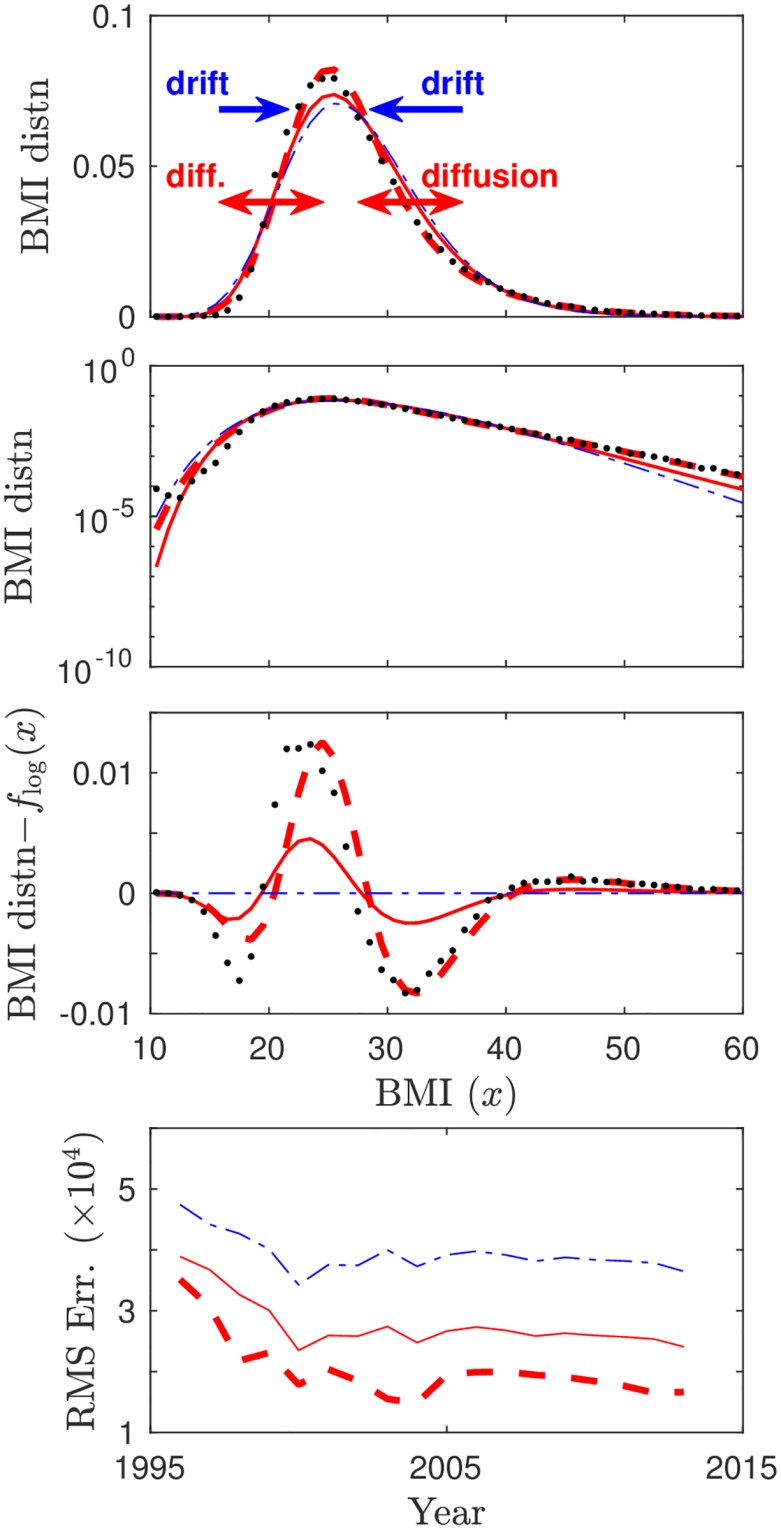
Results from fitting the 2011 NU empirical BMI distribution (black dots) to our predicted distributions $p_{eq}^{\left(0\right)}\left(x\right)$ (no social effects; red solid) and *p*_*eq*_(*x*) (with social effects; red dashed), and to a standard log-normal (blue dash-dotted) distribution. From top to bottom, the first panel illustrates how the BMI distribution results from a balance between drift and diffusion, and is right-skewed. The second panel shows the same BMI distributions in log scale to make tails more visible, and the third panel shows differences between the log-normal distribution as null-model and the other distributions. The second and third panels show that the $p_{eq}^{\left(0\right)}\left(x\right)$ (red solid) and *p*_*eq*_(*x*) (red dashed) distributions are more successful in fitting the empirical data than the commonly used log-normal distribution, both near the center of the distribution and in the high-BMI tail. This is confirmed in the bottom panel that shows the root mean-square error (RMSE) resulting from fitting NU data to BMI distributions in the range 1997–2014.

In Fig 3 we compare our new theoretical quasi-stationary BMI distributions with a candidate distribution function that is commonly used to describe right-skewed data (such as BMI distributions [8]): the log-normal probability distribution function

$$\begin{array}{c} f_{\text{log}}\left(x;\mu ,\sigma \right)=\frac{1}{x\sqrt{2\pi \sigma^{2}}}\exp[-\frac{{\left(\log x-\mu \right)}^{2}}{2\sigma^{2}}]\;. \end{array}$$ (13)

Because our model assumes that parameters are constant over short time scales, we fit each year of empirical BMI distribution data separately from each other. For details on how we fit empirical BMI distributions, see Section S1.2 of S1 Appendix.

Fig 3 shows that our non-social model (two parameters) gives a better fit to empirical BMI distributions than the log-normal distribution (two parameters). Our social model (four parameters) has the best fit. These findings are confirmed for publicly available data from the NHANES [27] and BRFSS [13] surveys, see Fig B of S1 Appendix.

To investigate the importance of the social utility contribution to *a*(*x*) in Eq (2) we compute the relative likelihood ratios of all BMI distribution models using the Akaike Information Criterion (AIC) [39], which quantifies the trade-off between goodness-of-fit and model complexity (number of parameters). Table 1 indicates that our social model is a better fit to the data than the nonsocial model for data year 2011 when taking into account the number of parameters, especially for our large NU data set. For other data years than 2011 we obtain similar AIC results. This lends some support to the hypothesis that correlations in the behavior of individuals with similar BMI play a role in individual BMI dynamics. As discussed before, in our extended model the interaction term of *a*(*x*) in Eq (2) was included to represent social transmission [16, 17, 19], but it can more broadly be interpreted as a term that adds the effect of correlations in the behavior of individuals with similar BMI, such as may occur due to food insecurity [36, 37] or certain gene-environment interactions [38]. Our results thus appear to indicate that such correlations in the behavior of individuals with similar BMI may be important. However, the interaction term in Eq (2) is just one possible extension of the basic model, and it is possible that other unmodeled effects are equally or more important. Demonstrating social transmission in a more direct way would require data that includes information about peer BMI.

**Table 1:** Akaike Information Criterion test for model distributions fitted to 2011 empirical BMI distribution data in Fig 3 and Fig B of S1 Appendix. Relative likelihood ratio exp[(*AIC*_min_−*AIC*)/2] of non-social $p_{eq}^{\left(0\right)}\left(x\right)$, social *p*_*eq*_(*x*), and log-normal *f*_log_(*x*) models for 2011 NU, NHANES and BRFSS empirical BMI distributions.

Data	Relative Likelihood Ratio		
	$p_{eq}^{0}\left(x\right)$	*p*_*eq*_(*x*)	*f*_log_(*x*)
NU	< 10^−300^	1	< 10^−300^
NHANES	4.3 × 10^−5^	1	6.0 × 10^−34^
BRFSS	< 10^−300^	1	< 10^−300^

## Discussion

### A mechanism for right-skewed broadening of BMI distributions over time

Our findings on drift and diffusion in BMI dynamics (as in Fig 2), together with the associated mathematical model, offer a new and compelling mechanism to explain the observed right-skewness of BMI distributions [8, 10–12]: in essence, random fluctuations broaden the BMI distribution away from the set point, and the broadening is stronger on the high-BMI side because the random variations in BMI are proportional to BMI (Fig 2, red triangles). When explaining the right-skewness, there is thus no need to invoke *singular effects* such as the “runaway train” mechanism [11], in which high-BMI individuals become subject to a self-reinforcing cycle of weight gain. In fact, we demonstrate that high-BMI individuals on average *strongly decrease* their weight year-over-year (Fig 2, blue dots). However, they are subject to large-amplitude fluctuations (with both positive and negative signs) that broaden the BMI distribution more on the high-BMI side than the low-BMI side. In S1 Appendix Section S1.2.6, we explain similarly that increasing fluctuations over time also explain the broadening of BMI distributions over time especially on the high-BMI side [10, 12]. In particular, S1 Appendix Section S1.2.6 precisely quantifies the ongoing right-skewed broadening of BMI distributions using expressions for the SD and skewness of our theoretical BMI distribution of Eq (12) (see Table B in S1 Appendix), and the observed evolution of the mean, the SD, and the ratio of the rate parameters *k*_*I*_/*k*_*b*_, see Fig 1 and Fig C in S1 Appendix. Essentially, the observed growth in average BMI over time (Fig 1) implies more fluctuations since fluctuations are proportional to BMI (Fig 2, red triangles), and more fluctuations mean a broadening of the distribution. We emphasize, however, that whereas these changes in BMI distribution over time are reflected in our model through changes in the fitted values of the model parameters, our model is about aggregate effects on the whole population, with parameters fitted to BMI data, and our model does not identify or specify individual root causes of the recent increases observed in population-average BMI.

Overall, the fluctuations in BMI represent the *aggregate effect* of natural variations in diet and physical activity, and perturbations that result from factors ranging from biology to psychology to social phenomena [8, 10, 12, 40], which may indeed include genetic effects [10, 14] and self-reinforcing weight gain such as in the “runaway train” [11]. The essential reason for the right-skewness (and its increase over time) can be traced back to the proportionality of BMI fluctuations to BMI, in the balance between drift and diffusion: individuals are subject to multifactorial perturbations and, for biological reasons, high-BMI individuals tend to lose or gain more weight due to these perturbations [8, 12, 28]. The fluctuations, thus, broaden the distribution more on the high-BMI side.

### Implications for public health interventions

Our results offer new insight into a mechanism that causes ongoing right-skewed broadening over time of BMI distributions in high-income societies. The mechanism we identified does not discriminate by socioeconomic and demographic factors, which is consistent with recent findings [10]. It will be important to reconcile the new understanding offered by this mechanism with the qualitative theories that are currently being debated to explain the right-skewed broadening over time [10–12, 14]. Specifically, our results indicate that, as the population BMI average increases over time [41, 42], the whole population is sensitive to increasing BMI fluctuations (Fig 2, red triangles). These fluctuations ultimately broaden the distribution (especially on the high-BMI side) and increase the high-BMI segment of the population. This adds justification to interventions that target the whole population [6, 7]. On the other hand, we demonstrate and quantify that high-BMI individuals are particularly at risk for large fluctuations that may result from multifactorial perturbations (Fig 2, red triangles), and our results confirm that reducing these fluctuations by discouraging perturbations such as yo-yo dieting [43] should be another focus of intervention.

More broadly, our results establish a form of statistical mechanics for human weight change. Analogous to drift-diffusion processes in physics and finance [31, 44], our empirical findings and mathematical model provide a new understanding of the role of drift and diffusion mechanisms in the dynamics of BMI distributions in human populations.

## Supporting information

S1 Video Animation of empirical BMI distributions drawn from BRFSS data (1987–2013). (Red dots) Empirical probability density function computed from BRFSS data year-by-year. (Solid red line) Result of fitting empirical data to non-social model, i.e. Eq (12), year-by-year. (Dashed blue line) Result of fitting empirical data to solution of full Fokker-Planck equation (see Section S1.2.3 of S1 Appendix for details).
(AVI)

S1 Appendix This Supporting information file contains further information on data, methods, and the data and code files (see [20] and S1 Matlab Code, respectively) that we make available with this manuscript, followed by Figs A–D, and Tables A and B. Numbers for equations, figures and tables that are not prefixed by S refer to the main text of the paper.
(PDF)

S1 Matlab Code The results presented in this paper were generated using these Matab m-files. (ZIP)
