# Supplementary material for: The statistical mechanics of human weight change
Source: PLoS One. 2017 Dec 18;12(12):e0189795. doi: 10.1371/journal.pone.0189795 (PMC5734703; doi:10.1371/journal.pone.0189795)
Supplement: S1 Appendix — Numbers for equations, figures and tables that are not prefixed by S refer to the main text of the paper. (PDF) [file pone.0189795.s002.pdf]

Supporting Information S1 - Appendix

The statistical mechanics of human weight change

John C. Lang, Hans De Sterck, and Daniel M. Abrams

This Supporting Information file contains further information on data, methods, and the data and code files (see [20] and S1 Matlab code, respectively) that we make available with this manuscript, followed by Supporting Figs **A**–**D**, and Supporting Tables **A** and **B**. Numbers for equations, figures and tables that are not prefixed by S refer to the main text of the paper.

S1.1 Additional Details on BMI Data Sets

S1.1.1 Northwestern Medicine Medical Records

The NU data set consists of medical records from the Northwestern Medical system of hospitals and clinics, i.e., patients of Northwestern Memorial Hospital, Lake Forest Hospital, and 15 other Chicago area locations: Bucktown (1776 N. Milwaukee Avenue, Chicago, Illinois 60647), Deerfield (350 S. Waukegan Road Suites 100, 150 and 200, Deerfield, Illinois 60015), Delano Court (in the Roosevelt Collection, 1135 S. Delano Court Suite A201, Chicago, IL 60605), Evanston (1704 Maple Avenue Suites 100 and 200, Evanston, Illinois 60201), Glenview (2701 Patriot Boulevard, Glenview, Illinois 60026), Grayslake (1475 E. Belvidere Road, Pavilion C Suite 385, Grayslake, IL 60030), Highland Park (600 Central Avenue Suite 333, Highland Park, Illinois 60035), Libertyville (1800 Hollister Drive Suite 102, Libertyville, Illinois 60048), Lakeview (1333 W. Belmont Avenue Suites 100 and 200, Chicago, Illinois 60657), Loop 1 (20 S. Clark Street Suite 1100, Chicago, Illinois 60603), Loop 2 (111 W. Washington St. Suite 1801, Chicago, Illinois 60602), River North (635 N. Dearborn Street Suite 100, Chicago, Illinois 60654), Sauganash (4801 W Peterson, Suite 406, Chicago, IL 60646), Skokie (10024 Skokie Blvd Suite 304, Skokie, IL, 60077), and SoNo (South of North Avenue, 1460 N. Halsted Street Suites 203, 502, and 504, Chicago, Illinois 60642).

We note that the NU data set may contain multiple measurements per individual per year. In that case the BMI for individual i in year t is calculated using the average weight of individual i in year t and the average height of individual i taken over all years.

For the purpose of computing year-over-year BMI changes, the Northwestern Medicine medical record contains measurements for 329,453 distinct individuals whose BMI can be calculated at at least two time points (1,017,518 BMI differences in total).

S1.1.2 National Health and Nurtition Examination Survey

The National Health and Nutrition Examination Survey (NHANES) refers to a series of studies designed to collect a representative sample of health and nutrition data for both adults and children (approximately 5,000 individuals total per year) in the United States. NHANES data are available for survey years 1999-2000, 2001-2002, . . . , 2013-2014. Directly measured BMI data are available from measurements taken during a physical exam. These data are used to compute empirical BMI distributions for each survey year. In addition, during an interview individuals were asked to self-report their current weight and height, as well as their weight from the preceding year. These measurements allow us to calculate self-reported change in BMI over the year preceding the interview. Note: we only use NHANES data for individuals 18 years or older at the time of the survey.

NHANES data are available from the NHANES website

`http://www.cdc.gov/nchs/nhanes/nhanes_questionnaires.htm`.

Directly measured BMI measurements are given by the variable BMXBMI. Self-reported BMI measurements are calculated from the variables WHD010 (self-reported height at time of interview) and WHD020 (self-reported weight at time of interview). Self-reported change in BMI over the year preceding the interview are calculated from self-reported BMI and from variables WHD010 and WHD050 (self-reported weight one year prior to interview).

Data were downloaded directly from the Centers for Disease Control and Prevention (CDC) website as ".XPT" files (in SAS format) and imported into Matlab. The variable BMXBMI is found in data files with names starting with "BMX", the variables WHD010, WHD020, and WHD050 are found in data files with names starting with "WHQ", the variable RIDAGEYR is found in data files with names starting with "DEMO", and the SEQN variable is found in all data files. File names are completed by adding the suffix ".XPT" for survey year 1999-2000, "_B.XPT" for survey year 2001-2002, "_C.XPT" for survey year 2003-2004, etc...

S1.1.3 Behavioral Risk Factor Surveillance System

The Behavioral Risk Factor Surveillance System (BRFSS) refers to a series of telephone surveys designed to collect a representative sample of health data for adults (aged 18 years or older) in the United States. BRFSS data are available for survey years 1984, 1985, . . . , 2013. We note that prior to 2011 BRFSS surveys were conducted over land lines only, whereas from 2011 onward BRFSS methodology has been modified to include cell phones as well. We also note that many states did not participate in early BRFSS surveys. Therefore, for the purposes of this study we only consider surveys from 1987 (the first year where a majority of states participated in the BRFSS) onwards. The number of individual records for each BRFSS survey increases from approximately 50,000 in 1987, to approximately 135,000 in 1997, to more than 400,000 from 2007 onward. For each BRFSS survey we extract the BMI of each individual surveyed and use this data to compute the empirical BMI distribution for that year. We note that since these data are gathered using telephone interviews, the weight and height measurements (used in calculating BMI) are all self-reported, in contrast to the NHANES and NU data sets. Also in contrast to NHANES and NU data sets, the BRFSS data does not provide sufficient data for us to compute the change in individuals' BMI over time.

BRFSS data are available from the BRFSS website

`http://www.cdc.gov/brfss/annual_data/annual_data.htm`.

BRFSS surveys record BMI measurements in variable _BMI for survey years 1984-1999, _BMI2 for survey years 2000-2002, _BMI3 for survey year 2003, _BMI4 for survey years 2004-2010, and _BMI5 for survey years 2011 onwards. Data were downloaded directly from the CDC website as ".XPT" files (in SAS format) and imported into Matlab. File names for BRFSS survey data for years 1978–2010 start with "CDBRFS", while file names for BRFSS survey data for years 2011–2013 start with "LLCP". File names are completed by adding the suffix "87.XPT' for year 1987, "88.XPT" for year 1988, etc...

S1.2 Additional Details on Methods

S1.2.1 Properties of $p_{eq}^{(0)}(x; k_0, x^\star)$ (Eq (12))

The properties of $p_{eq}^{(0)}(x; k_0, x^\star)$ (Eq (12)) listed in Table **B** can be derived as follows. We note that for any population the BMI distribution must be strictly contained in the interval $[0, \infty)$. This implies that $p_{eq}(0) = 0$ and that $\lim_{x\to\infty} p_{eq}(x) = 0$. Assuming that $b(0) = 0$ (which holds for our model, see Eq (10)), it follows that integrating Eq (11) with vanishing temporal derivative yields

$$0 = -p_{eq}(x)a(x) + \frac{1}{2}\frac{d}{dx}\left[p_{eq}(x)b^2(x)\right] + \underbrace{p_{eq}(0)a(0) - \frac{1}{2}\frac{d}{dx}\left[p_{eq}(x)b^2(x)\right]_{x=0}}_{=0} , \tag{S1.1}$$

which has the solution

$$p_{eq}(x) = \xi \exp\left(2\int_0^x \frac{a(\tilde{x}) - b(\tilde{x})b'(\tilde{x})}{b^2(\tilde{x})} d\tilde{x}\right) , \tag{S1.2}$$

where ξ is a normalization constant such that $\int_0^\infty p_{eq}(x)dx = 1$. When $a(x) = k_I(x^\star - x)$ (no social effects, i.e., $k_S = 0$ in Eq (2)) and $b(x) = \sqrt{k_b}\, x$, we can re-arrange Eq (S1.1) to yield

$$\frac{dp_{eq}^{(0)}}{dx}(x) = 2\frac{k_I(x^\star - x) - k_b x}{k_b x^2} p_{eq}^{(0)}(x) ,$$

which implies that $p_{eq}^{(0)}(x)$ is a single peaked probability distribution whose mode is given by the expression $x^\star \frac{k_I}{k_b}/(\frac{k_I}{k_b}+1)$. (The mode of a continuous random variable with probability density function $f(x)$ is $\text{argmax}_x f(x)$.) We can also re-arrange Eq (S1.1) to yield

$$x p_{eq}^{(0)}(x) = -\frac{k_b}{k_I}\frac{d}{dx}\left[\frac{x^2}{2} p_{eq}^{(0)}(x)\right] + x^\star p_{eq}^{(0)}(x) ,$$

which implies that

$$\langle x \rangle = \int_0^\infty x p_{eq}^{(0)}(x)dx = -\frac{k_b}{k_I}\underbrace{\int_0^\infty \frac{d}{dx}\left[\frac{1}{2}x^2 p_{eq}^{(0)}(x)\right]dx}_{=0} + x^\star \underbrace{\int_0^\infty p_{eq}^{(0)}(x)dx}_{=1} = x^\star .$$

Multiplying Eq (S1.1) by x and re-arranging yields

$$x^2 p_{eq}^{(0)}(x) = -\frac{1}{2}\frac{k_b}{k_I}\frac{d}{dx}\left[x^3 p_{eq}^{(0)}(x)\right] + \frac{1}{2}\frac{k_b}{k_I}x^2 p_{eq}^{(0)}(x) + x x^\star p_{eq}^{(0)}(x) ,$$

which implies

$$\begin{aligned}\langle x^2 \rangle &= \int_0^\infty x^2 p_{eq}^{(0)}(x)dx \\ &= -\frac{1}{2}\frac{k_b}{k_I}\int_0^\infty \frac{d}{dx}\left[x^3 p_{eq}^{(0)}(x)\right]dx + \frac{1}{2}\frac{k_b}{k_I}\int_0^\infty x^2 p_{eq}^{(0)}(x)dx + x^\star \int_0^\infty x p_{eq}^{(0)}(x)dx \\ &= \frac{1}{2}\frac{k_b}{k_I}\langle x^2 \rangle + x^{\star 2} .\end{aligned}$$

Re-arranging now yields $\langle x^2 \rangle = 2x^{\star 2}\frac{k_I}{k_b}/(2\frac{k_I}{k_b} - 1)$. We note that we require $\langle x^2 \rangle \geq 0$, i.e. that $2k_I/k_b > 1$. We also note that this condition is satisfied by all empirical BMI distributions in the NU, NHANES and BRFSS data sets. Similarly, multiplying Eq (S1.1) by x^2, re-arranging, integrating, and solving for $\langle x^3 \rangle$, yields $\langle x^3 \rangle = \frac{k_I}{k_b}/(\frac{k_I}{k_b} - 1)\langle x^2 \rangle x^\star$.

The mean, variance, skewness, and mode skewness can now be computed using the following relations to the mode and the first three moments.

$$\begin{aligned}
\text{mean} &= \langle x \rangle \\
\text{variance} &= \left\langle x^2 \right\rangle - \langle x \rangle^2 \\
\text{skewness} &= \frac{\left\langle x^3 \right\rangle - 3\langle x \rangle (\text{variance}) - \langle x \rangle^3}{(\text{variance})^{\frac{3}{2}}}, \text{ and} \\
\text{mode skewness} &= \frac{\text{mean} - \text{mode}}{(\text{variance})^{\frac{1}{2}}}\ .
\end{aligned}$$

Remark: When $x^\star$ is distributed according to the probability density function $f(\cdot)$ then mean, mode, standard deviation, skewness, and mode skewness are given by the same expressions as in Table **B**, where $x^\star$ is replaced by $\langle x^\star \rangle = \int x^\star f(x^\star) dx^\star$. This is the case because when $x^\star$ is distributed according to the probability density function $f(\cdot)$ then the distribution function for BMIs in the population is given by

$$\hat{p}_{eq}^{(0)}(x) = \int p_{eq}^{(0)}(x) f(x^\star) dx^\star.$$

So, for example,

$$\langle x \rangle = \int \int x p_{eq}^{(0)}(x) f(x^\star) dx^\star dx = \int \underbrace{\int x p_{eq}^{(0)}(x) dx}_{=x^*} f(x^\star) dx^\star = \langle x^\star \rangle\ .$$

S1.2.2 Solving Eq (S1.2) for $p_{eq}(x)$ (social model, $k_S \neq 0$)

In the case of the social model ($k_S \neq 0$ in Eq (2)), Eq (S1.2) does not provide a closed-form solution for the equilibrium distribution. However, the stationary solution to Eq (11) is given implicitly by Eq (S1.2), i.e., Eq (S1.2) becomes

$$p_{eq}(x) \propto p_{eq}^{(0)}(x; k_I/k_b, x^\star) \exp\left(2\frac{k_S}{k_b} \int_0^x \int_0^\infty \frac{\phi_{\tilde{x},\sigma}(\hat{x})(\hat{x} - \tilde{x}) \left(1 - \frac{1}{2}\frac{(\hat{x}-\tilde{x})^2}{\sigma^2}\right) p_{eq}(\hat{x})}{\tilde{x}^2} d\hat{x} d\tilde{x} \right)\ ,$$

where we consider the continuum limit and the discrete sum in Eq (2) has been replaced by an integral over the population with distribution $p_{eq}(x)$. In order to solve for $p_{eq}(x)$ numerically, we implement the following iterative scheme in which we discretize the iterative approximations $p_{eq}^{(i)}(x)$ and approximate the double integral numerically.

$$\begin{aligned}
p_{eq}^{(n+1)}(x) &= p_{eq}^{(n+1)}(x; k_I/k_b, x^\star, k_S/k_b, \sigma) \\
&\propto p_{eq}^{(0)}(x; k_I/k_b, x^\star) \exp\left(2\frac{k_S}{k_b} \int_0^x \int_0^\infty \frac{\phi_{\tilde{x},\sigma}(\hat{x})(\hat{x} - \tilde{x}) \left[1 - \frac{1}{2}\frac{(\hat{x}-\tilde{x})^2}{\sigma^2}\right] p_{eq}^{(n)}(\hat{x})}{\tilde{x}^2} d\hat{x} d\tilde{x} \right)\ .
\end{aligned} \tag{S1.3}$$

Let $m = 181$, $\Delta z = 0.5$, and and $\forall i = 1, 2, \ldots, m : z_i = 10 + (i-1)\Delta z$. We set

$$p_{eq}^{(0)}(z_i) = \frac{z_i^{-2(k_I/k_b+1)} \exp\left(-2\frac{k_I}{k_b}\frac{x^\star}{z_i}\right)}{\sum_{j=1}^m z_j^{-2(k_I/k_b+1)} \exp\left(-2\frac{k_I}{k_b}\frac{x^\star}{z_j}\right) \Delta z}\ .$$

We then set

$$p_{eq}^{(n+1)}(z_i) = p_{eq}^{(0)}(z_i) \exp\left(2\frac{k_S}{k_b}\sum_{k=1}^{i}\sum_{j=1}^{m}\frac{1}{2}\left(1+\mathbb{I}_{\{k<i\}}\right)\frac{\phi_{z_k,\sigma}(z_j)(z_j-z_k)\left(1-\frac{1}{2}\frac{(z_j-z_k)^2}{\sigma^2}\right)p_{eq}^{(n)}(z_j)}{z_k^2}\Delta z^2\right)$$

where $\mathbb{I}_{\{X\}} = 1$ if X is true and $\mathbb{I}_{\{X\}} = 0$ otherwise, and where we terminate the iterative process once

$$\left\|\frac{p_{eq}^{(n+1)} - p_{eq}^{(n)}}{m}\right\|_2 = \sqrt{\sum_{i=1}^{m}\left(\frac{p_{eq}^{(n+1)}(z_i) - p_{eq}^{(n)}(z_i)}{m}\right)^2} < 10^{-12} \ .$$

S1.2.3 Fitting distribution functions to empirical BMI distributions

Suppose that $f(x;\theta)$ is a probability density function with parameters θ. We fit $f(x;\theta)$ to empirical BMI data measurements $\{x_i\}_{i=1}^N$ using the principle of maximum likelihood parameter estimation. Specifically, we set

$$\hat{\theta} = \underset{\theta}{\mathrm{argmax}} \prod_{i=1}^{N} f(x_i;\theta),$$

where $\mathcal{L}_f(\theta|x) = \prod_{i=1}^N f(x_i;\theta)$ is called the likelihood function. In Matlab we perform this optimization using the Matlab function *fminsearch* to solve the equivalent optimization problem

$$\hat{\theta} = \underset{\theta}{\mathrm{argmin}} -\log(\mathcal{L}_f(\theta|x)) = \underset{\theta}{\mathrm{argmin}} -\sum_{i=1}^{N}\log\left[f(x_i|\theta)\right]$$

We note that we compute a separate set of parameters for each year of BMI data.

Remark: In fitting empirical BMI distributions to the equilibrium distribution of our model, i.e. either the non-social model in Eq (12) or the social model in Eq (S1.3) (see Fig. 3), we made the assumption that the empirical distribution was close to the equilibrium distribution. We argued that this assumption was justified because parameter values in our model drift on a time scale that is slower than individual equilibration times. Alternatively, we could have parametrized the parameters

$$\begin{aligned} x^\star(t) &= x_0^\star + x_1^\star(t-t_0) + \ldots + x_n^\star(t-t_0)^n/n! \\ k_I(t) &= k_{I,0} + k_{I,1}(t-t_0) + \ldots + k_{I,n}(t-t_0)^n/n!, \text{ and} \\ k_b(t) &= k_{b,0} + k_{b,1}(t-t_0) + \ldots + k_{b,n}(t-t_0)^n/n! \ , \end{aligned}$$

and fit the solution to the full Fokker-Planck equation, i.e. Eq (11), to the empirical BMI distributions (again using the principle of maximum likelihood parameter estimation). We note that we have not adopted this strategy in the main text because (a) it is very computationally intensive and (b) the result does not differ significantly from when we fit empirical BMI distributions to the equilibrium distribution of our model. To illustrate this point, we compare fitting BRFSS BMI distributions to the non-social model in Eq (12) and to the solution of the full Fokker-Planck equation with $n = 1$, as described above, see S1 Video.

S1.2.4 Estimating parameters $(\hat{k}_I, \hat{x}^\star, \hat{k}_S, \hat{\sigma})$ in $a(x)$ (Eq (2)) and $\widehat{\sqrt{k_b}}$ in $b(x)$ (Eq (10))

In this section we describe how we estimate the parameters for the drift and diffusion curves (solid black) in Fig. 2, fitted to the individual-level NU and NHANES data for all available years. The fitted parameters are presented in Table **A**.

Consider individual i from survey year $t = t_1$ with BMI measurements at times t_1 and $t_2 = t_1 + \Delta t$, i.e. with BMI measurements $x_i(t_1)$ and $x_i(t_2)$. We denote the change in BMI by $\Delta x_i(t_t) = x_i(t_2) - x_i(t_1)$. For $\epsilon > 0$ we define

$$\hat{a}(x_i(t);\epsilon) = \frac{\sum_{j:|x_j(t)-x_i(t)|<\epsilon} \frac{\Delta x_j(t)}{\Delta t}}{N(x_i(t),\epsilon)}, \text{ and} \tag{S1.4a}$$

$$\hat{b}(x_i(t);\epsilon) = \sqrt{\frac{\sum_{j:|x_j(t)-x_i(t)|<\epsilon} \frac{\Delta x_j^2(t)}{\Delta t}}{N(x_i(t),\epsilon)} - \Delta t \left[\frac{\sum_{j:|x_j(t)-x_i(t)|<\epsilon} \frac{\Delta x_j(t)}{\Delta t}}{N(x_i(t),\epsilon)}\right]^2}\ , \tag{S1.4b}$$

respectively, where $N(x_i(t),\epsilon)$ is the number of individuals j with $|x_j(t) - x_i(t)| < \epsilon$, i.e.,

$$N(x_i(t),\epsilon) = \sum_{j:|x_j(t)-x_i(t)|<\epsilon} 1\ .$$

We note that in order to reduce computation time we do not compute Eqs (S1.4a) and (S1.4b) for each individual i separately. Instead, we compute Eqs (S1.4a) and (S1.4b) on the grid $\{10, 10.1, 10.2, \ldots, 100\}$ and then evaluate $\hat{a}(x_i(t);\epsilon)$ and $\hat{b}(x_i(t);\epsilon)$ using linear interpolation.

To estimate $\sqrt{k_b}$ we compute $\hat{b}(x_i(t);\epsilon)$ from BMI data and regress it on $x_i(t)$. To estimate the remaining parameters we define the objective function

$$\mathcal{S}(k_I, x^\star, k_S, \sigma; \epsilon) = \sqrt{\frac{\sum_{i,t}\left[\hat{a}(x_i(t);\epsilon) - a(x_i(t))\right]^2}{\sum_{i,t} 1}}$$

and solve the optimization problem

$$\left(\hat{k}_I, \hat{x}^\star, \hat{k}_S, \hat{\sigma}\right) = \underset{(k_I, x^\star, k_S, \sigma)}{\operatorname{argmin}}\ \mathcal{S}(k_I, x^\star, k_S, \sigma; \epsilon), \tag{S1.5}$$

where we have suppressed the dependence of $(\hat{k}_I, \hat{x}^\star, \hat{k}_S, \hat{\sigma})$ on ϵ for convenience of notation. Recall from Eq (9) that

$$a(x_i(t)) = k_I(x^\star - x_i(t)) + k_S \frac{du_S}{dx_i}(x_i(t)),$$

where

$$\frac{du_S}{dx_i}(x_i(t)) = \frac{1}{N(t)} \sum_{j=1}^{N(t)} \phi_{x_i(t),\sigma}(x_j(t))(x_j(t) - x_i(t)) \left(1 - \frac{1}{2}\frac{(x_j(t) - x_i(t))^2}{\sigma^2}\right),$$

and where $N(t)$ is the number of observations in survey year t. Observe that, for fixed σ, the objective function $\mathcal{S}(k_I, x^\star, k_S, \sigma; \epsilon)$ is the objective function for the linear regression of $\hat{a}(x_i(t);\epsilon)$ on $-x_i(t)$, $\frac{du_S}{dx_i}(x_i(t);\sigma)$, and a constant. It follows, therefore, that there is a unique $(\tilde{k}_I(\sigma), \tilde{x}^\star(\sigma), \tilde{k}_S(\sigma))$ that solves

$$\left(\tilde{k}_I(\sigma), \tilde{x}^\star(\sigma), \tilde{k}_S(\sigma)\right) = \underset{(k_I, x^\star, k_S)}{\operatorname{argmin}}\ \mathcal{S}(k_I, x^\star, k_S, \sigma; \epsilon)\ , \tag{S1.6}$$

and that can be computed using linear regression. Solving the optimiation problem in Eq (S1.5) is now reduced to a one dimensional problem, i.e., we solve

$$\hat{\sigma} = \underset{\sigma}{\operatorname{argmin}}\ \mathcal{S}\left(\tilde{k}_I(\sigma), \tilde{x}^\star(\sigma), \tilde{k}_S(\sigma), \sigma\right)$$

and set $(\hat{k}_I, \hat{x}^\star, \hat{k}_S) = (\tilde{k}_I(\hat{\sigma}), \tilde{x}^\star(\hat{\sigma}), \tilde{k}_S(\hat{\sigma}))$.

We estimate the parameters k_I and $x^\star$ with $k_S = 0$ by regressing $\hat{a}(x_i(t);\epsilon)$ on $-x_i(t)$ and a constant. Note that when $k_S = 0$ the parameter σ is undetermined.

We note that the methodology presented in this section can only be applied to NHANES and NU BMI data, because these are the only data sets that have information on how individuals' BMI changes over time. We are able to compute $\Delta x_i(t)$ for individuals i in the NHANES data set with self-reported weights WHD010 (current, i.e. at time t_2) and WHD050 (one year prior to survey, i.e. at time t_1), and with self-reported height WHD020. For convenience we set $t = 1999$ for the 1999-2000 NHANES survey, $t = 2001$ for the 2001-2002 NHANES survey, etc... We note that for the NHANES data $\Delta t = 1$. For NU data we also consider $\Delta t = 1$, i.e., we consider individuals for whom we can compute BMI in two consecutive years. NU data for individuls in consecutive years exists for years

$$t \in \{1996, \ldots, 2013\}.$$

As above, if multiple weight measurements are present in year t we calculate the BMI for that year using the average weight in year t, whereas if multiple heights measurements are present then we calculate BMI using the average height (where the average is taken over all years). We note that for both data sets we use $\epsilon = \frac{1}{2}$ to compute Eqs (S1.4a) and (S1.4b).

All computations are performed in Matlab. Regressions are performed using the Matlab function *regress*. Optimization are performed using the Matlab function *fminsearch*.

S1.2.5 Akaike Information Criterion

We give a brief overview of maximum likelihood estimation and the Akaike Information Criterion.

S1.2.5.1 Maximum Likelihood Estimation

Suppose that we have independently and identically distributed (IID) data $\{x_i\}_{i=1}^N$ that are drawn from the unknown probability distribution $p(x)$. Suppose also that we are attempting to model the unknown probability distribution $p(x)$ by the family of parametric probability distribution functions $\{f(x|\theta)\}_\theta$, i.e. our goal is to find the $\hat{\theta}$ such that, of all the functions in $\{f(x|\theta)\}_\theta$, $f(x|\hat{\theta})$ is the "best" approximation to $p(x)$. The maximum likelihood estiamtor (MLE) $\hat{\theta}$ is the parameter that maximizes the likelihood function $\mathcal{L}_f(\theta|x)$, i.e.

$$\hat{\theta} = \underset{\theta}{\operatorname{argmax}} \underbrace{\prod_{i=1}^{N} f(x_i|\theta)}_{=\mathcal{L}_f(\theta|x)} . \tag{S1.7}$$

We note that the relative likelihood function

$$r = \frac{\mathcal{L}_f(\theta|x)}{\mathcal{L}_f(\hat{\theta}|x)}$$

is interpreted as follows: $f(x|\theta)$ is r times as likely as $f(x|\hat{\theta})$ to be the "best" approximation to $p(x)$. In this case, "best" is in the context of maximizing the likelihood function.

S1.2.5.2 Akaike Information Criterion

The Akaike Information Criterion (AIC) is a generalization of the principle of maximum likelihood estimation. An equivalent formulation of the MLE given in Eq. (S1.7) is given by maximizing the average log-likelihood function $\mathcal{S}_N(f(\cdot|\theta))$, i.e.

$$\hat{\theta} = \underset{\theta}{\operatorname{argmax}} \frac{1}{N} \log\left(\mathcal{L}_f(\theta|x)\right) = \underset{\theta}{\operatorname{argmax}} \underbrace{\frac{1}{N} \sum_{i=1}^{N} \log(f(x_i|\theta))}_{=\mathcal{S}_N(f(\cdot|\theta))} .$$

It can be shown that the mean log-likelihood function $\mathcal{S}_N(f(\cdot|\theta))$ converges with probability 1 to

$$\mathcal{S}(p; f(\cdot|\theta)) = \int p(x)\log(f(x|\theta))dx.$$

From this quantity we define the Kullback-Leibler mean information for the discrimination between $p(x)$ and $f(\cdot|\theta)$

$$I(p; f(\cdot|\theta)) = \mathcal{S}(p;p) - \mathcal{S}(p; f(\cdot|\theta)),$$

which can be shown to be non-negative, with $I(p; f(\cdot|\theta)) = 0 \iff f(x|\theta) = p(x)$ almost everywhere. Roughly speaking, $I(p; f(\cdot|\theta))$ can be interpreted as the amount of information lost when $f(\cdot|\theta)$ is used to approximate $p(x)$. This quantity induces a natural model selection criterion, i.e. we select the model that minimizes $I(p; f(\cdot|\theta))$.

Remarks:

1. $\mathcal{S}(p; f(\cdot|\theta))$ can be approximated by $\mathcal{S}_N(f(\cdot|\theta))$, which can be computed from the data without knowing the "true" distribution $p(x)$.
2. Setting $\hat{\theta} = \text{argmin}_\theta\, I(p; f(\cdot|\theta))$, equivalently $\hat{\theta} = \text{argmax}_\theta\, \mathcal{S}(p; f(\cdot|\theta)) \approx \text{argmax}_\theta\, \mathcal{S}_N(f(\cdot|\theta))$ recovers the MLE.

The key observation for the establishment of the AIC criterion is that the quantity $I(p; f(\cdot|\theta))$ can be approximated as follows. Suppose that the true model is $p(x) = f(x|\theta_0)$ for some $\theta_0 \in \Theta$ and suppose that $\hat{\theta}$ is the MLE for the model restricted to some k-dimensional subspace $\Theta' \subset \Theta$, i.e.

$$\hat{\theta} = \underset{\theta\in\Theta'\subset\Theta}{\text{argmax}}\, \mathcal{L}_f(\theta|x).$$

Then it can be shown that

$$\mathbb{E}\left[2N\ I(p; f(\cdot|\hat{\theta}))\right] = \mathbb{E}\left[2N\ I(f(\cdot|\theta_0); f(\cdot|\hat{\theta}))\right] = c+2k-2\sum_{i=1}^{N}\log\left(f(x_i|\hat{\theta})\right) = c+\underbrace{2k-2\log\left(\mathcal{L}_f(\hat{\theta}|x)\right)}_{=AIC(f(\cdot|\hat{\theta}))},$$

where c is a constant, k is the dimension of Θ' (i.e., the number of parameters in the model), and where $AIC(f(\cdot|\hat{\theta})) = 2k - 2\log\left(\mathcal{L}_f(\hat{\theta}|x)\right)$ is the Akaike Information Criterion (AIC). It follows that minimizing $I(p; f(\cdot|\theta))$ is equivalent to minimizing the AIC. A key point is that k is the number of parameters in the model, and the AIC deals with the trade-off between the goodness of fit of the model and the complexity (number of parameters) of the model.

We now would like to generalize the likelihood ratio introduced above. Suppose that we compute the AIC for two different models resulting in AIC values $AIC_1 = 2k_1 - 2\log(\mathcal{L}_1)$ and $AIC_2 = 2k_2 - 2\log(\mathcal{L}_2)$ with $AIC_1 < AIC_2$. Then the relative likelihood ratio

$$r = \exp\left(\frac{AIC_1 - AIC_2}{2}\right) = \exp(k_1 - k_2)\frac{\mathcal{L}_2}{\mathcal{L}_1}$$

is interpreted as follows: model 1 is r times as likely to be the "best" approximation to the true distribution than model 2. In this case, "best" is in the context of minimizing the AIC (i.e. minimizing the loss of information when using models 1/2 to approximate the "true" distribution $p(x)$).

S1.2.6 Ongoing Right-Skewed Broadening of the BMI Distribution over Time

Our results offer a mechanism to explain why BMI distributions continue to broaden over time, especially on the high-BMI side. Essentially, in the context of our findings the observed growth in average

BMI (Fig. 1) implies more fluctuations since fluctuations are proportional to BMI (Fig. 2, red triangles), and more fluctuations mean a broadening of the distribution. In addition we also consider the following effect. Recall that the constants k_I and k_b determine the rates in the set point drift term of Eq (2) and the diffusion amplitude of Eq (10), respectively. We observe a decrease over time of $k_0 = k_I/k_b$, which reflects a growing relative importance of fluctuations over drift (see Fig. **C**), and we will explain now that this also implies a broadening of the distribution, especially on the high-BMI side.

All these effects can be quantified precisely using expressions for the mean, mode, variance, skewness, and mode skewness of our theoretical BMI distribution Eq (12) (see Table **B**). The table expresses these quantities in terms of $x^\star$ and $k_0 = k_I/k_b$, see SM Section S1.2.1 for detailed calculations. Note that, in order for the variance to be non-negative we require that $2k_I/k_b - 1 > 0$. This condition is satisfied in all the empirical BMI distributions considered in this study.

As was shown in Fig. 1, BMI mean and SD have both steadily grown since at least 1987 while the obesity epidemic was running its course (with tempered growth in more recent years).

In terms of explaining why SDs of US BMI distributions continue to increase over time and why BMI distributions broaden, the formula for the SD in our theoretical BMI distribution of Eq (12), given by $x^\star/\sqrt{2k_0 - 1}$ (Table **B**), provides insight. SD increases proportionally with the mean BMI, and a decrease in k_0 (increasing importance of fluctuations) also implies an increase in SD. Intuitively, an increase in the mean BMI implies more fluctuations since fluctuations are proportional to BMI, and a decrease in k_0 (the relative importance of drift over fluctuations) also implies more fluctuations. These increasing fluctuations naturally broaden the BMI distribution over time.

The skewness of our theoretical BMI distribution Eq (12) is given by $2\sqrt{2k_0 - 1}/(k_0 - 1)$ (Table **B**). Note that positive values for skewness correspond to right-skewness. Fig. **C** shows that $k_0 = k_I/k_b$, which reflects the relative importance of drift over fluctuations, has steadily decreased over the course of the obesity epidemic, at least since 1987. This decrease is likely due in large part to an increase in the fluctuation proportionality constant $\sqrt{k_b}$ over time, which may plausibly be linked to the factors that have caused the increase in average BMI for the population over time, for example, an increase in average calorie intake or portion sizes over time. Indeed, if human BMIs are characterised by short-term fluctuations (Fig. 2, red triangles), one can expect these fluctuations to become larger when average calorie intake or portion sizes increase over time. Applying skewness formula $2\sqrt{2k_0 - 1}/(k_0 - 1)$ to the fitted values of k_0 in Fig. **C**, one finds, for example, that the skewness ≈ 0.77 for $k_0 \approx 15$ (for 1996), and skewness increases as k_0 decreases over time. This shows that our predicted BMI distribution naturally features right-skewness (essentially due to the fluctuations being larger on the high-BMI side), and that skewness increases over time (since k_0 decreases).

S1.3 Data and Code Files Made Available with this Manuscript

The following data and code files are made available as additional Supporting Information in a single zipped archive. All figures and tables of this paper are fully reproducible using these files.

S1.3.1 Matlab Code

The results presented in this paper were generated using the following three Matlab m-files.

1. *BMI_Master.m*: Executes files *fitBMIdistn.m*, *fitAB.m*, and *dpdt.m*, see below.
2. *fitBMIdistn.m*: Performs population-level calculations, i.e. fits nonsocial model $p_{eq}^{(0)}(x)$, social model $p_{eq}(x)$, and log-normal $f_{\log}$ distribution to empirical BMI distribution data.
3. *fitAB.m*: Performs individual-level calculations, i.e. computes coefficients $a(x)$ and $b(x)$ from year-over-year change in BMI data.
4. *dpdt.m*: Fit solution to the partial differential equation (non-social model) with parameters x^*, k_I, and k_b that vary linearly in time to empirical BMI distributions computed from BRFSS data.

These files are included in the Supporting Information file S1 Matlab code.

S1.3.2 NU, NHANES, and BRFSS BMI Data Files

The following files are used by the Matlab Code described in the previous section and have been deposited with Dryad [20].

1. NU data are stored in *NU.csv* comma separated values (CSV) format. This file contains five columns: year t, BMI in year t, BMI in year $t+1$, age in year t, and gender.
   - *Note*: When BMI in year $t+1$ is unavailable then the entry in the third column is -1.
2. NHANES data are either self-reported (used to calculate year-over-year change in BMI) or directly measured (used to compute BMI distributions) data. We associate NHANES data from survey 1999-2000 with $t = 1999$, from survey 2001-2002 with $t = 2001$, and so on.
   (a) Self-reported NHANES data are stored in *NHANES_SR.csv* in CSV format. This file contains five colums: year t, BMI in year $t-1$ (i.e. year prior to the interview), BMI in year t (i.e. at time of interview), age in year t, and gender.
      - *Note*: Because self-reported NHANES data are only used for individual-level computations, *NHANES_SR.csv* only records data from respondents who reported both (a) BMI at time of NHANES interview and (b) BMI one year prior to NHANES interview.

   (b) Directly measured NHANES data are stored in *NHANES_DM.csv* in CSV format. This file contains four columns: year t, BMI in year t, age in year t, and gender.
3. BRFSS data are stored in *BRFSS_BMI.csv* in CSV format. This file contains two columns: year t, BMI in year t.

Supplementary Figures (Figs A–D)

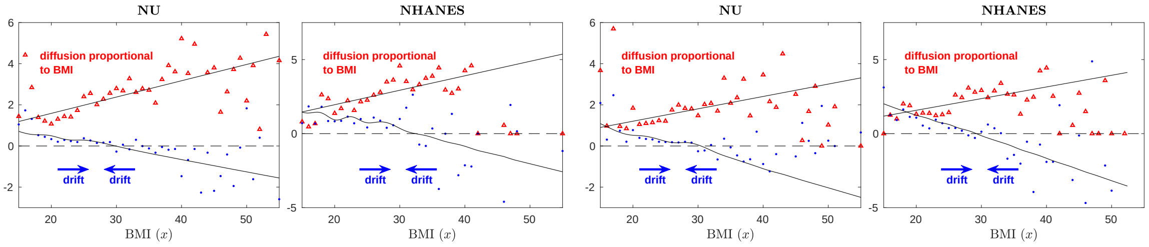

(a) Ages 18-28 (left: female, right: male)

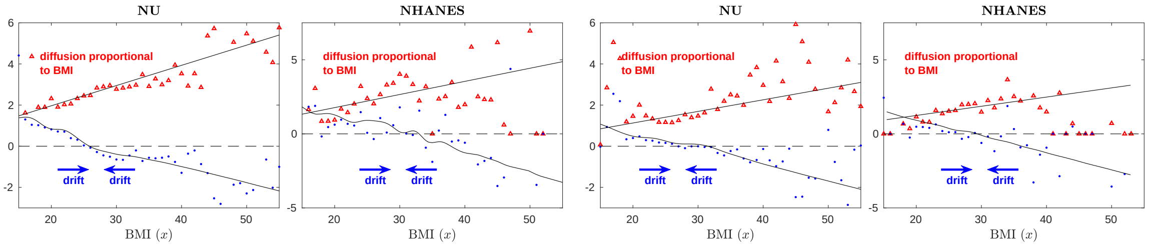

(b) Ages 28-38 (left: female, right: male)

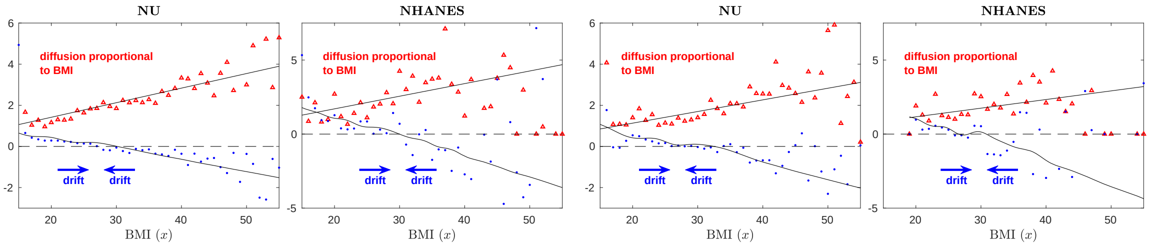

(c) Ages 38-48 (left: female, right: male)

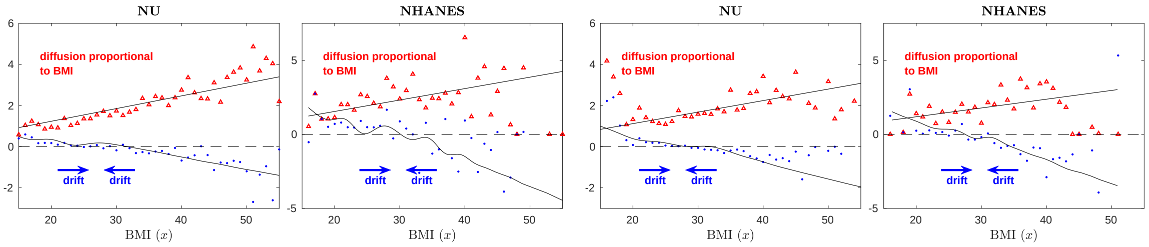

(d) Ages 48-58 (left: female, right: male)

Fig A: **Drift and diffusion in the short-term BMI dynamics of individuals in a human population, by age and gender, corresponding to Fig. 2 in the main text.** The figure shows the average annual change in the BMI of individuals (blue dots), and the standard deviation of the annual change in the BMI of individuals (red triangles), as a function of BMI, for data from our new large NU data set (initial exams occuring in 2011) and from the publicly available NHANES survey data set (measurements recorded in 2011-2012 survey). These results confirm that BMI dynamics feature a *drift* towards a set point, and a *diffusion* that is proportional to the BMI. The black curves are the curves of best fit to our mathematical models for the drift term (Eq (2), including social effects) and for the diffusion amplitude (Eq (10)), as discussed in the Methods and mathematical models section.

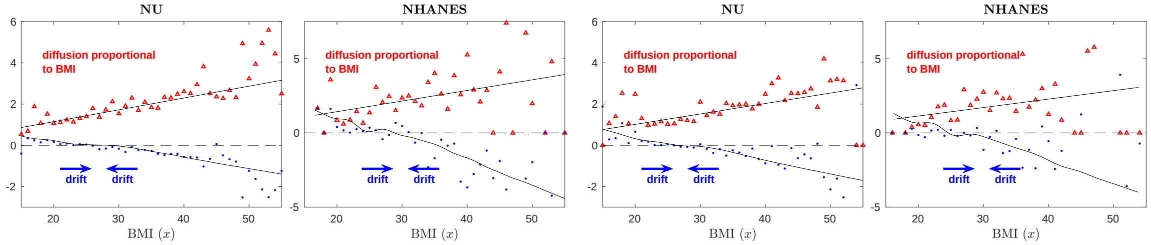

(e) Ages 58-68 (left: female, right: male)

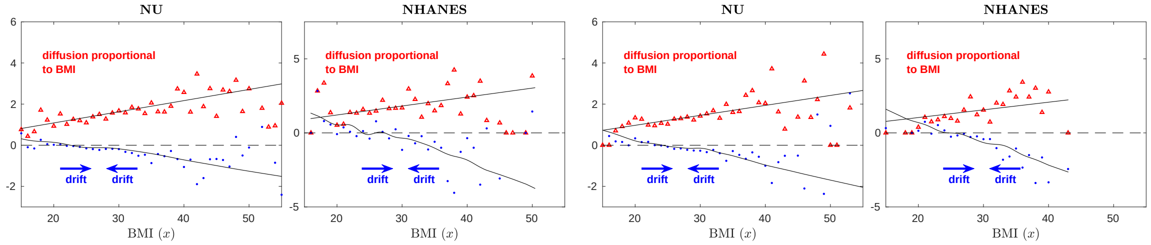

(f) Ages 68-78 (left: female, right: male)

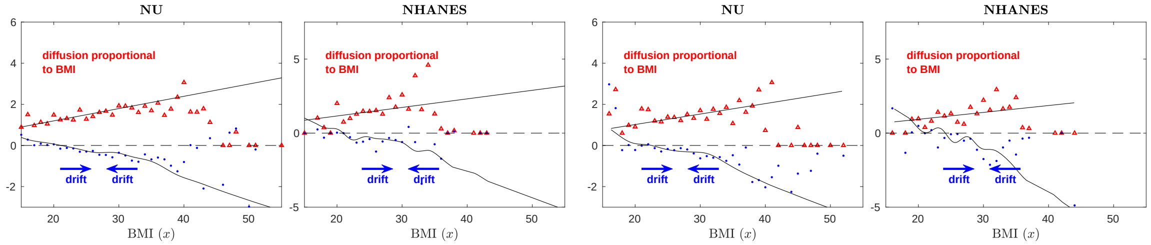

(g) Ages 78 and over (left: female, right: male)

Fig A: **Drift and diffusion in the short-term BMI dynamics of individuals in a human population, by age and gender, corresponding to Fig. 2 in the main text.** The figure shows the average annual change in the BMI of individuals (blue dots), and the standard deviation of the annual change in the BMI of individuals (red triangles), as a function of BMI, for data from our new large NU data set (initial exams occuring in 2011) and from the publicly available NHANES survey data set (measurements recorded in 2011-2012 survey). These results confirm that BMI dynamics feature a *drift* towards a set point, and a *diffusion* that is proportional to the BMI. The black curves are the curves of best fit to our mathematical models for the drift term (Eq (2), including social effects) and for the diffusion amplitude (Eq (10)), as discussed in the Methods and mathematical models section.

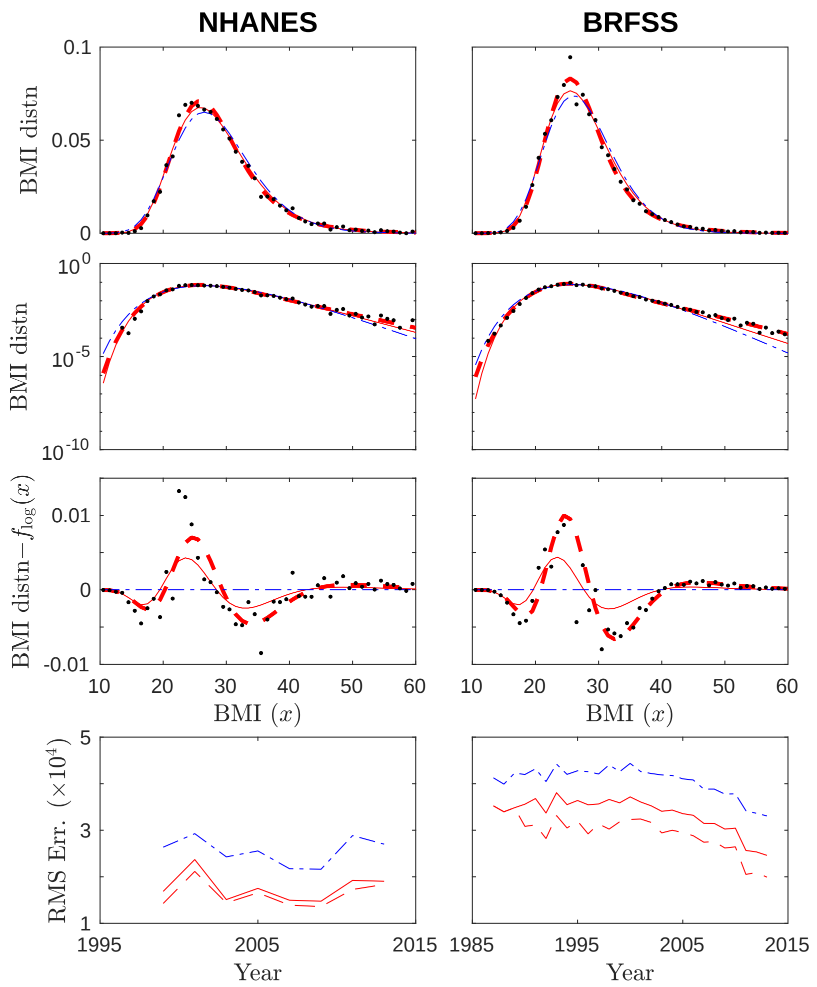

Fig B: **Results from fitting the 2011 NHANES and BRFSS empirical BMI distributions (black dots) to predicted distributions $p_{eq}^{(0)}(x)$ (no social effects; red solid) and $p_{eq}(x)$ (with social effects; red dashed), and to a standard log-normal (blue dash-dotted) and skew-normal (green dotted) distributions.** The top panels illustrate that the BMI distribution is right-skewed. The second-line panels show the BMI distributions in log scale, and the third-line panels show the difference between the log-normal distribution as null-model and the other distributions. The second-line and third-line panels show clearly that the new $p_{eq}^{(0)}(x)$ and $p_{eq}(x)$ distributions are more successful in fitting the empirical data than the commonly used log-normal and skew-normal distributions. The non-social $p_{eq}^{(0)}(x)$ (red solid), and, in particular the social $p_{eq}(x)$ (red dashed), are a much better fit to the empirical data than the two standard distributions, both in the central part of the distribution (third-line panels) and in the high-BMI tail (second-line panels). Note that the improvement of the social model is less pronounced in the NHANES data, which is likely due to the very small sample size in the NHANES data. This is confirmed in the bottom panels that show the root mean-square error (RMSE) for the data over the full range of years. Overall, the NHANES and BRFSS results are fully consistent with the observations in the main paper for the more extensive NU data.

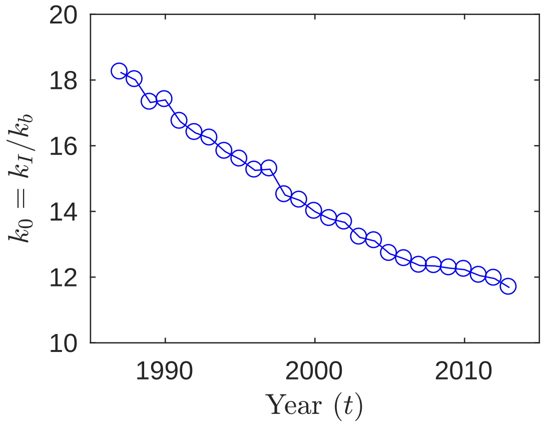

Fig C: **Fitted parameter $k_0 = k_I/k_b$ for each available year of the BRFSS survey data (nonsocial model).** The relative importance of drift over fluctuations has steadily decreased in the course of the obesity epidemic.

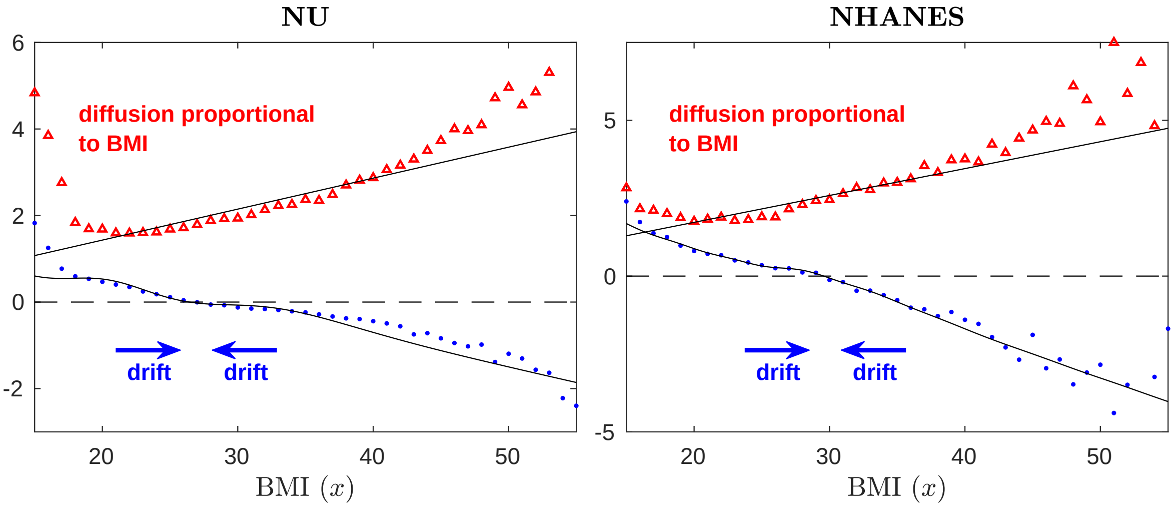

Fig D: **Drift and diffusion in the short-term BMI dynamics of individuals in a human population, for the entire data set over all years.** This Figure repeats Fig 2 (with 2011-2012 data) from the main text, but now for the entire data set over all years. The average annual change in the BMI of individuals (blue dots), and the standard deviation of the annual change in the BMI of individuals (red triangles), are shown as a function of BMI, for the NU and NHANES data sets over all years. These results confirm, for the entire data set, the nearly linear relations for the annual change and its standard deviation that were identified in Fig 2 for data years 2011-2012. Due to increased data size, the curves for the entire data set are less noisy. We can observe that the standard deviation appears to grow faster than linear for large BMIs greater than about 45, both for the NU patient data and the NHANES population data (which is still noisy for the largest BMIs). The black curves are the curves of best fit for all data years to our mathematical models for the drift term (Eq (2), including social effects) and for the diffusion amplitude (Eq (10)), as discussed in the Methods and mathematical models section.

Tables A and B

Table A: **Parameter estimates for the drift and diffusion curves (solid black) in Fig. 2, fitted to the individual-level NU and NHANES data for all available years.** We estimate k_I, $x^\star$, k_S, and σ in the drift term $a(x)$ (Eq (2)) for both the nonsocial ($k_S = 0$) and social ($k_S \neq 0$) models. We also estimate $\sqrt{k_b}$ in the diffusion amplitude $b(x)$ (Eq (10)). The fitting procedure is described in Section S1.2.4.

Data	Model	Parameters				L_2-Error	$\sqrt{k_b}$	L_2-Error
		$\hat{k}_I$	$\hat{x^\star}$	$\hat{k}_S$	$\hat{\sigma}$			
NHANES	nonsocial	0.127	28.0	0	–	0.566	0.084	0.497
	social	0.149	28.0	25.2	2.08	0.545		
NU	nonsocial	0.059	28.0	0	–	0.374	0.071	0.461
	social	0.069	28.0	9.4	3.44	0.365		

Table B: **Properties of the BMI equilibrium distribution** $p_{eq}^{(0)}(x; k_0, x^\star)$**, Eq** (12) **(**$k_0 = k_I/k_b$**; no social interaction).** Increase in $x^\star$ over time implies increase in SD (consistent with Fig. 1); our model essentially explains this using the observation that fluctuations increase proportional to BMI (Fig. 2). Similarly, SD and right-skewness also increase when k_0 decreases over time (as shown in Fig. **C**), i.e., when fluctuations become relatively more important than drift.

Property	Value
Mean	$x^\star$
Mode	$k_0 x^\star/(k_0 + 1)$
Standard Deviation	$x^\star/\sqrt{2k_0 - 1}$
Skewness	$2\sqrt{2k_0 - 1}/(k_0 - 1)$
Mode Skewness	$\sqrt{2k_0 - 1}(k_0 + 1)$
